# European primary forest database v2.0

**DOI:** 10.1038/s41597-021-00988-7

**Published:** 2021-08-17

**Authors:** Francesco Maria Sabatini, Hendrik Bluhm, Zoltan Kun, Dmitry Aksenov, José A. Atauri, Erik Buchwald, Sabina Burrascano, Eugénie Cateau, Abdulla Diku, Inês Marques Duarte, Ángel B. Fernández López, Matteo Garbarino, Nikolaos Grigoriadis, Ferenc Horváth, Srđan Keren, Mara Kitenberga, Alen Kiš, Ann Kraut, Pierre L. Ibisch, Laurent Larrieu, Fabio Lombardi, Bratislav Matovic, Radu Nicolae Melu, Peter Meyer, Rein Midteng, Stjepan Mikac, Martin Mikoláš, Gintautas Mozgeris, Momchil Panayotov, Rok Pisek, Leónia Nunes, Alejandro Ruete, Matthias Schickhofer, Bojan Simovski, Jonas Stillhard, Dejan Stojanovic, Jerzy Szwagrzyk, Olli-Pekka Tikkanen, Elvin Toromani, Roman Volosyanchuk, Tomáš Vrška, Marcus Waldherr, Maxim Yermokhin, Tzvetan Zlatanov, Asiya Zagidullina, Tobias Kuemmerle

**Affiliations:** 1 German Centre for Integrative Biodiversity Research (iDiv) - Halle-Jena-Leipzig, Puschstrasse 4, 04103 Leipzig, Germany; 2 Martin-Luther-Universität Halle-Wittenberg, Institut für Biologie, Am Kirchtor 1, 06108 Halle, Germany; 3 Humboldt-Universität zu Berlin, Geography Department, Unter den Linden 6, 10099 Berlin, Germany; 4 Frankfurt Zoological Society, Bernhard-Grzimek-Allee 1, 60316 Frankfurt, Germany; 5 NGO “Transparent World”, Rossolimo str. 5/22, building 1, 119021 Moscow, Russia; 6 EUROPARC-Spain/Fundación Fernando González Bernáldez. ICEI Edificio A. Campus de Somosaguas, E28224 Pozuelo de Alarcón, Spain; 7 The Danish Nature Agency, Gjøddinggård, Førstballevej 2, DK-7183 Randbøl, Denmark; 8 Sapienza University of Rome, Department of Environmental Biology, P.le Aldo Moro 5, 00185 Rome, Italy; 9 Réserves Naturelles de France, La Bourdonnerie, Dijon cedex, 21000 France; 10 PSEDA-ILIRIA. Forestry department, Tirana, 1000 Albania; 11 Centre for Applied Ecology “Professor Baeta Neves” (CEABN), InBIO, School of Agriculture, University of Lisbon, Tapada da Ajuda, 1349‐017 Lisbon, Portugal; 12 Parque Nacional de Garajonay. Avda. V Centenario, edif. Las Creces, local 1, portal 3, 38800 San Sebastian de La Gomera, Tenerife, Spain; 13 University of Torino, Department DISAFA L.go Paolo Braccini 2, Grugliasco, 10095 Italy; 14 Forest Research Institute, Vassilika, 57006 Thessaloniki, Greece; 15 Centre for Ecological Research, Institute of Ecology and Botany, Alkotmány u. 2-4., 2163 Vácrátót, Hungary; 16 Faculty of Forestry, University of Agriculture in Krakow, aleja 29-Listopada 46, 31-415 Krakow, Poland; 17 Latvian State Forest Research Institute “Silava”, Rigas street 111, Salaspils, LV-2169 Latvia; 18 Institute for Nature Conservation of Vojvodina Province, Radnička 20a, Novi Sad, 21000 Serbia; 19 University of Tartu, Institute of Ecology and Earth Sciences, Vanemuise 46, EE-51014 Tartu, Estonia; 20 Centre for Econics and Ecosystem Management, Faculty of Forest and Environment, Eberswalde University for Sustainable Development, Alfred-Möller-Str. 1, 16225 Eberswalde, Germany; 21 Université de Toulouse, INRAE, UMR DYNAFOR, 24 Chemin de Borde-Rouge Auzeville CS 52627, Castanet-Tolosan, 31326 France; 22 CRPF-Occitanie, antenne de Tarbes, place du foirail, 65000 Tarbes, France; 23 Mediterranean University of Reggio Calabria, Agraria Department, Loc. Feo di Vito, 89122 Reggio Calabria, Italy; 24 University of Novi Sad, Institute of Lowland Forestry and Environment, Antona Cehova 13d, Novi Sad, 21102 Serbia; 25 World Wide Fund for nature (CEE), Lunga street 190, Brasov, 500051 Romania; 26 Northwest German Forest Research Institute, Department Forest Nature Conservation, Professor-Oelkers-Straße 6, 34346. Hann, Münden, Germany; 27 Asplan Viak A.S.Kjörboveien 20, postboks 24, N-1300 Sandvika, Norway; 28 University of Zagreb, Faculty of Forestry, Svetosimunska cesta 25, 10000 Zagreb, Croatia; 29 Czech University of Life Sciences, Faculty of Forestry and Wood Sciences, Kamýcka cesta 1176, CZ-16521 Praha6-Suchdol, Czech Republic; 30 PRALES, Odtrnovie 563, SK-01322 Rosina, Slovakia; 31 Vytautas Magnus University, K. Donelaičio g. 58, LT-44248 Kaunas, Lithuania; 32 University of Forestry, Dendrology Department, bulevard “Sveti Kliment Ohridski” 10, 1756 Sofia, Bulgaria; 33 Slovenia Forest Service, Department for forest management planning, Vecna pot 2, 1000 Ljubljana, Slovenia; 34 Centre for Applied Ecology “Professor Baeta Neves” (CEABN), InBIO, School of Agriculture, University of Lisbon, Tapada da Ajuda 1349‐017, Lisbon, Portugal; 35 Greensway AB, Ulls väg 24A. 756 51, Uppsala, Sweden; 36 Freelance forest expert and book author, Vienna, Austria; 37 Ss. Cyril and Methodius University in Skopje, Hans Em Faculty of Forest Sciences, Landscape Architecture and Environmental Engineering, Department of Botany and Dendrology, P.O. Box 235, MK-1000 Skopje, North Macedonia; 38 Swiss Federal Research Institute for Forest, Snow and Landscape Research WSL, Forest Resources and Management, Zürcherstrasse 111, 8903 Birmensdorf, Switzerland; 39 University of Novi Sad, Institute of Lowland Forestry and Environment, Antona Cehova 13d, Novi Sad, 21000 Serbia; 40 Department of Forest Biodiversity, University of Agriculture, Kraków, Poland; 41 University of Eastern Finland, School of forest Sciences, Yliopistokatu 7, 80100 Joensuu, Finland; 42 Agricultural University of Tirana, Forestry Department, Kodër Kamëz, SH1, 1029 Tirana, Albania; 43 World Wide Fund for nature (DCP) Ukraine, Mushaka 48, Lviv, 79011 Ukraine; 44 Ecosphera NGO, Kapushans’ka 82a, Uzhhorod, 88000 Ukraine; 45 Silva Tarouca Research Institute, Department of Forest Ecology, Lidická 25/27, 602 00 Brno, Czech Republic; 46 Centre for Econics and Ecosystem Management, Faculty of Forest and Environment, Eberswalde University for Sustainable Development, Alfred-Möller-Str. 1, 16225 Eberswalde, Germany; 47 Institute of Experimental Botany of the National Academy of Sciences of Belarus, Laboratory of Productivity & Stability of Plant Communities, 220072 Academicheskaya St. 27, Minsk, Belarus; 48 Institute of Biodiversity and Ecosystem Research, Bulgarian Academy of Sciences, 2 Gagarin Street, 1113 Sofia, Bulgaria; 49 Saint-Petersburg State University, Department of Vegetation Science, University Embankment, 7/9, St Petersburg, 199034 Russia; 50 Humboldt-Universität zu Berlin, Geography Department & Integrative Research Institute on Transformation in Human-Environment Systems, Unter den Linden 6, 10099 Berlin, Germany

**Keywords:** Biodiversity, Forest ecology, Conservation biology

## Abstract

Primary forests, defined here as forests where the signs of human impacts, if any, are strongly blurred due to decades without forest management, are scarce in Europe and continue to disappear. Despite these losses, we know little about where these forests occur. Here, we present a comprehensive geodatabase and map of Europe’s known primary forests. Our geodatabase harmonizes 48 different, mostly field-based datasets of primary forests, and contains 18,411 individual patches (41.1 Mha) spread across 33 countries. When available, we provide information on each patch (name, location, naturalness, extent and dominant tree species) and the surrounding landscape (biogeographical regions, protection status, potential natural vegetation, current forest extent). Using Landsat satellite-image time series (1985–2018) we checked each patch for possible disturbance events since primary forests were identified, resulting in 94% of patches free of significant disturbances in the last 30 years. Although knowledge gaps remain, ours is the most comprehensive dataset on primary forests in Europe, and will be useful for ecological studies, and conservation planning to safeguard these unique forests.

## Abstract (summary)

**Table:** 

Measurement(s)	Geographic Distribution
Technology Type(s)	digital curation
Factor Type(s)	country
Sample Characteristic - Environment	primary forest
Sample Characteristic - Location	Europe

Machine-accessible metadata file describing the reported data: 10.6084/m9.figshare.14540625

## Background & Summary

The importance of primary forests is widely recognized^1,2^. First, they provide refuge to forest biodiversity^3^, and act as a buffer to species loss in human-dominated landscapes^4^. Second, primary forests play an important role in climate change mitigation. At the local scale, they buffer the adverse effects of increasing temperature on understory biodiversity, as they often have cooler forest-floor summer temperatures compared to secondary forests^5^. At the global scale they contribute to climate stability by storing large quantities of carbon, both in the biomass and in soils^1,6,7^. Third, primary forests often serve as a reference for developing close-to-nature forest management, or for benchmarking restoration efforts^8^. Finally, these forests are an irreplaceable part of our natural heritage, shape the cultural identities of local communities, and have a high intrinsic value^9^.

In Europe, as in many human-dominated regions, most forests are currently managed^10^, often with increasing harvest intensities^11,12^. As a result, despite the general trend of increasing total forest area, primary forests are scarce and continue to disappear^13^. For instance, Romania hosts some of the largest swaths of primary forest in Central Europe and faced a sharp increase in logging rates since 2000. This has resulted in significant primary forest loss, even within protected areas^13–15^. In Poland, the iconic Białowieża Forest was recently in the spotlight after the controversial decision from the Polish National Forest Holding, now nullified by the Court of Justice of the European Union^16^, to implement salvage logging followed by tree planting after a bark beetle outbreak^17^. Widespread loss of primary forests also occurred in Ukraine^18^, Slovakia^19^, or in the boreal North, e.g., in the Russian North-West, where 4.6 Mha of primary forest were lost since 2001^13,20^. Effective protection of Europe’s primary forests is therefore urgently needed^21^.

In the newly released ‘Biodiversity Strategy for 2030’, the European Commission emphasized the need to define, map, monitor and strictly protect all of the EU’s remaining primary forests^2^. Reaching these objectives requires complete and up-to-date data on primary forests’ location and protection status. Such data could inform both policy making and conservation planning, as well as research, for instance by highlighting areas where primary forests are either scarce, or poorly studied. Yet, many data gaps remain on the location and conservation status of EU’s primary forests^21,22^. Only a few countries conducted systematic, on-the-ground inventories^19,23^. For most countries data are either only available for a few well-studied forests^24–26^, or are limited to the distribution of potential (=unconfirmed) primary forests, typically predicted statistically or via remote sensing^27–29^. Despite past efforts for harmonizing data^24,30^, only recently has the first map of primary forests been released for Europe^31,32^ together with a first assessment of their conservation status^21^.

In a previous effort, we assembled a first European Primary Forest database (EPFD v1.0) that included 32 local-to-national datasets, plus data from a literature review and a survey, resulting in the mapping of a total of ~1.4 Mha of primary forest^31^. This was only about one fifth of the estimated 7.3 Mha of undisturbed forest still occurring in Europe, excluding Russia^10^. Also, most of the data collected in our v1.0 database were not open-access, and could thus not be used without the explicit consent of their respective copyright holders.

**Fig. 1:**
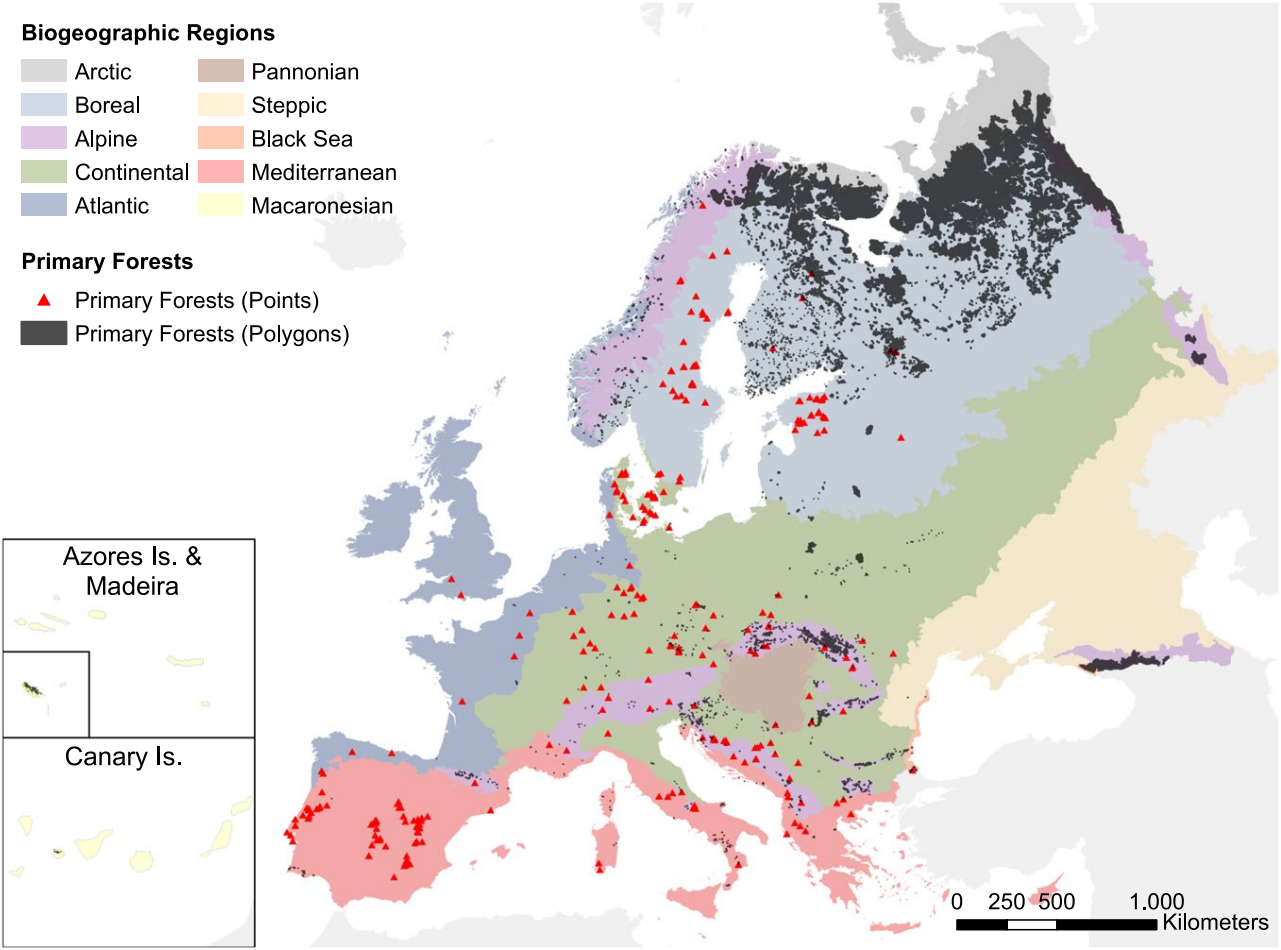
Overview of the primary forest patches contained in the EPFD v2.0. Both points and polygons were magnified to improve visibility.

**Fig. 2:**
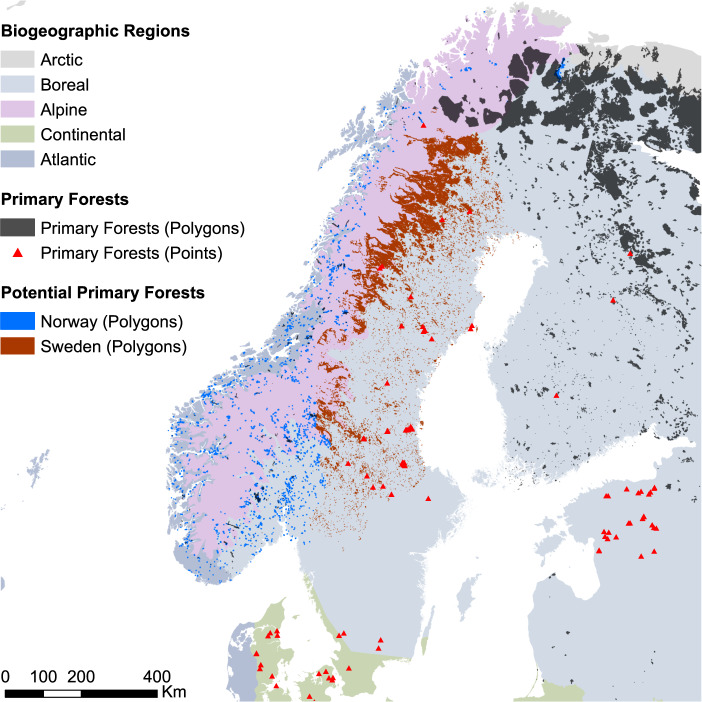
Overview of the maps of potential primary forests of Sweden and Norway.

Here, we build on those efforts to progress further towards a complete map of Europe’s primary forests. First, we secured permission from all data holders to release all data with open-access. Second, we aggregated and harmonized 16 additional regional-to-continental spatial datasets to now cover a total of 48 independent datasets. The EPDF v2.0 contains 18,411 non-overlapping primary forest patches (plus 299 point features) covering an area of 41.1 Mha (37.4 Mha in European Russia alone; Fig. 1) across 33 countries (Table 1)^33^. Key improvements of this new database include (a) filling major regional gaps, including European Russia, the Balkan Peninsula, the Pyrenees and the Baltic region, (2) mapping potential primary forests for Sweden and Norway (additional 16,311 polygons and 2.5 Mha - Fig. 2), two key regions where complete inventories are currently unavailable, and (3) an update of our literature review to January 2019.

**Table 1:** Summary of primary forest data across European countries.

Country	Num. features (Polygons\ Points)	Tot. estimated area (1,000 ha)	Sources (Dataset IDs)
Albania	13\6	13.36	0, 1, 47, 54
Austria	34\2	1.46	9, 35, 49
Belarus	3\0	188.29	46
Bosnia and Herzegovina	4\12	4.1	0, 2, 50, 53
Bulgaria	483\2	56.77	0, 3, 4, 35
Croatia	45\3	6.24	0, 5, 9
Czechia	86\10	9.07*	0, 6, 9
Denmark	0\24	1.68	7
Estonia	0\29	0.05*	0, 8
Finland	1,008\3	2,817.36*	0, 12, 38, 39
France	106\7	10.86*	0, 13, 14, 35, 37
Germany	25\21	13.65*	0, 9, 15, 35
Greece	5\2	1.75*	0, 16
Italy	86\12	6.84*	0, 18, 35, 55
Latvia	3\0	4.79	40
Lithuania	20\0	32.05	19
Moldova	0\1	0.03	35
Montenegro	2\0	2.85	2, 50
Netherlands	3\0	0.08	36
North Macedonia	5\1	0.81	1, 20
Norway	240\1	280.05*	0, 21, 36, 43
Poland	66\5	21.15*	0, 22, 35
Portugal	32\21	15.75*	23, 24
Romania	3,571\6	59.11*	0, 1, 25, 32, 33, 35
Russian Federation	3,082\3	37,417.69*	0, 51
Serbia	14\4	7.78	0, 35, 36, 44, 45
Slovakia	290\4	10.98	0, 9, 26
Slovenia	170\1	9.53	0, 27
Spain	44\58	9.4*	0, 41, 52
Sweden	0\51	32.81*	0, 29, 35
Switzerland	5\5	2.29	0, 30, 35
Ukraine	8,966\3	97.8*	0, 1, 32
United Kingdom	0\2	0.1	9
Total	18,411\299	41,136.53*	

Dataset IDs correspond to those in Online-only Table 1. * Some point features have no information on forest patch area.

## Methods

### Primary forest definition

Defining primary forests is controversial, and a range of different definitions have been put forward over the years^22^. In this paper, as in our previous work, we follow the FAO definition that defines primary forests^34^ as “*naturally regenerated forest of native tree species, where there are no clearly visible indications of human activities and the ecological processes are not significantly disturbed*”.

We operationalized this definition using the framework proposed by Buchwald^35^, where ‘primary forest’ is used as an umbrella term to include forests with different levels of naturalness, such as primeval, virgin, near‐virgin, old‐growth and long‐untouched forests. Based on this framework, a forest qualifies as primary if the signs of former human impacts, if any, are strongly blurred due to decades (at least 60–80 years) after the end of forest management^35^. This time limit, however, depends on how modified the forest was at the starting point, and only applies in the case of traditional management, such as patch felling, partial coppicing, or selective logging. Stands regenerating naturally after a clear cut would therefore require a longer time period to be considered a primary forest (i.e., 60–80 years plus the length of a typical rotation cycle). Our definition of primary forests, therefore, does not imply that these forests were never cleared or disturbed by humans. We consider this is in line with the Convention of Biological Diversity (CBD, https://www.cbd.int/forest/definitions.shtml), acknowledging that the concept of primary forests has a different connotation in Europe than in the rest of the world.

Finally, our collection of primary forests includes mainly old-growth, late-successional forests, but also some early seral stages and young forests that originated after natural disturbances and natural regeneration, without subsequent management. In case of large primary forest tracts (>250 ha), our polygons can also locally include land not covered by trees.

### Data collection

To create the EPFD v2.0, we first expanded and updated the literature review on primary forests we had originally carried out for EPFD v1.0^31^, which only considered the period 2000–2017, and excluded European Russia. Specifically, we added all scientific studies published between January 2000 and January 2019 for Russia, and those published in 2017–2019 for the rest of Europe. We identified relevant publications in the ISI Web of Knowledge using the search terms “(primary OR virgin OR old‐growth OR primeval OR intact) AND forest*” in the title field. Based on our own interpretation of commonly used forest terms, we deliberately excluded terms such as “unmanaged” (meaning: not under active management), “ancient” (never cleared for agriculture) or “natural” (stocked with naturally regenerated native trees). These terms indicate conditions that are necessary, but not sufficient for considering a forest as primary. Finally, we refined our search using geographical and subject filters. The literature search returned 129 candidate papers. After screening their content, we added 23 additional primary forest stands (10 in European Russia, 13 in the rest of Europe), from 13 studies (four from European Russia, and nine from the rest of Europe).

Building the EPFD v1.0^31^ involved reaching out to 134 forest experts. For v2.0 we contacted an additional 75 experts with knowledge on forests or forestry, and invited them to add spatially-explicit data on primary forests to our database. We focussed on experts from geographical regions poorly covered in v1.0. We received 56 answers, which led to the incorporation of 16 new datasets in our map. Given the context-dependency of definitions used in regional mapping projects, new datasets were only included if we could find an explicit equivalence between country-specific forest definitions and our definition framework^35^. This was done after discussing with data contributors the criteria and categories used for constructing their datasets, which we then mapped onto our definition framework. Depending on the datasets, these criteria included: (1) forest age or structural variables^19,23,36^, (2) legal designation^25^ or year since onset of protection^37^, (3) time since last anthropogenic disturbancee^38^, or (4) the lack of human impacts and infrastructures^39^.

We integrated all data into a geodatabase, which contains primary forests either as polygons (if information on the forest boundary was available) or point locations (when having only an approximate centre location). We set 0.5 hectares as minimum mapping unit, although only a few of the datasets already contained in v1.0 contained polygons smaller than 2 ha (i.e., the minimum mapping unit originally used). If available, we included a set of basic descriptors for each patch: name, location, naturalness level (based on^35^), extent, dominant tree species, disturbance history and protection status. In total, our map harmonizes 48 regional-to-continental datasets of primary forests (Online-only Table 1). All data is open-access^33^, except for three datasets that we kept confidential, either for conservation or copyright reasons. These datasets are: ‘Hungarian Forest Reserve monitoring’ (ID 17, custodian: Ferenc Horváth); ‘Ancient and Primeval Beech Forests of the Carpathians and Other Regions of Europe’^40,41^ (ID 34, copyright: UNESCO), and ‘Potential OGF and primary forest in Austria’ (ID 48, custodian: Matthias Schickhofer). Additional non-open access polygons also exist for the dataset ‘Strict Forest Reserves in Switzerland’ (ID 30, custodian: Jonas Stillhard). These data are here referred to for transparency, but are neither included in the statistics and summaries reported here, nor in any of the remote-sensing analysis below.

### Post-processing

To provide common descriptions for all features contained in the geodatabase, we integrated the basic descriptors detailed above with a range of attributes derived by intersecting all polygons or points of primary forests with layers of: 1) biogeographical regions, 2) protected areas, 3) forest type, and 4) forest cover.

We used the map of biogeographical regions^42^ to assign each primary forest point or polygon to one of the following ten classes: 1. Alpine, 2. Arctic, 3. Atlantic, 4. Black Sea, 5. Boreal, 6. Continental, 7. Macaronesia, 8. Mediterranean, 9. Pannonian, 10. Steppic. Similarly, we derived information on protection status and time since onset of protection for each primary forest polygon or point based on the World Database on Protected Areas (WDPA - https://www.protectedplanet.net). We simplified the original IUCN classification to three classes: 1. strictly protected – (IUCN category I); 2. protected – (IUCN categories II-VI + not classified); 3. not protected. This is a conservative aggregation recognizing the fact that, in certain contexts, logging and salvage logging are allowed inside national parks, at least in the buffer zone. In case of polygons, we considered a primary forest patch as protected if > 75% of its surface was within a WDPA polygon. When better information on the protection status of a forest patch was available directly from data contributors, we gave priority to this source. We also assigned each primary forest polygon or point to one of the forest categories defined by the European Environmental Agency^43^. The spatial information was derived by simplifying the map of Potential Vegetation types for Europe^44^, after creating an expert-based cross-link table^21^, which ties together forest categories and potential vegetation types reported in Table 4.1 from^43^. After excluding forest plantations, the remaining 13 categories comprised: 1. Boreal forest; 2. Hemiboreal forest and nemoral coniferous and mixed broadleaved-coniferous forest; 3. Alpine coniferous forest; 4. Acidophilous oakwood and oak-birch forest; 5. Mesophytic deciduous forest; 6. Lowland to submountainous beech forest; 7. Mountainous beech forest; 8. Thermophilous deciduous forest; 9. Broadleaved evergreen forest; 10. Coniferous forests of the Mediterranean, Anatolian and Macaronesian regions; 11. Mire and swamp forest; 12. Floodplain forest; 13. Non-riverine alder, birch or aspen forest. For each primary forest polygon (but not for points), we reported the two most common forest categories. Finally, we extracted for each primary forest polygon the actual share covered by forest. We did this, because larger primary forest polygons in high naturalness classes can encompass land temporarily or permanently not covered by trees. We used a tree cover density map for the year 2010 for these regions from^45^. All post-processing was performed in R (v3.6.1)^46^.

### Potential primary forests of Sweden and Norway

**Fig. 3:**
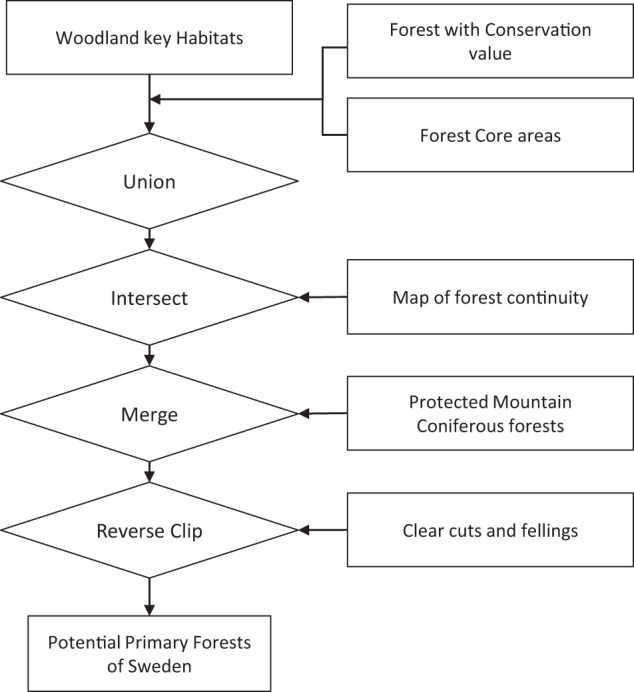
Workflow and data sources for the map of potential primary forests in Sweden. Data on woodland key habitats derive from^60^ (see also: https://www.skogsstyrelsen.se); forest with conservation value from^61,62^, forest core areas from^63^, continuity forests from^64,65^, protected mountain coniferous forests from^66^, clear cuts and fellings from https://www.skogsstyrelsen.se.

For Sweden and Norway, where abundant geographic information was available on forest distribution, we created maps of potential (but so far unconfirmed) primary forests. For Sweden, we derived a workflow to create a map of potential primary forests as detailed in Fig. 3. This yielded 14,300 polygons covering a total area of 2.4 Mha.

For Norway, even though we were able to include two datasets of confirmed primary forests, additional primary forest is expected to exist. Therefore, we derived a map of potential primary forests, based on the “*Viktige Naturtyper”* dataset from the Norwegian Environment Agency^47^, which maps different habitat types of high conservation value both inside and outside forested areas. We extracted all polygons larger than 10 ha classified as “*old forest types”* (=“gammelskog”), i.e., forests that have never been clearcut and are in age classes of 120 years or older. This yielded 2,103 polygons covering a total area of 0.1 Mha.

Importantly, these layers were neither directly integrated in our composite map, nor used to calculate country level statistics as they only represent a first approximation of the primary forest situation in these countries, so far without ground validation. Yet, we included these layers in our geodatabase with the goal of directing future ground-based mapping efforts.

## Data Records

The EPFD v2.0^33^ is composed of 48 individual datasets (Online-only Table 1) and the two layers of potential primary forests for Sweden and Norway. We integrated the 48 datasets into two composite feature classes, after excluding all duplicated\overlapping polygons across individual datasets.

1. *EU_PrimaryForests_Polygons_OA_v20*

- Composite feature class combining the forest patches classified as “primary forest” based on polygon data sources described in Online-only Table 1
- Data type: Polygon Feature Class

2) *EU_PrimaryForests_Points_OA_v20*

- Composite feature class combining forest locations classified as “primary forest”, based on point data sources described in Online-only Table 1. Only points not overlapping with polygons in (1) reported.
- Data type: Point Feature Class

The individual datasets are also included in the geodatabase, inside the feature dataset ‘*European_PrimaryForests’*. The whole database is stored in Figshare (10.6084/m9.figshare.13194095.v1)^33^. The file format is ESRI personal geodatabase (.mdb). Each feature class in the geodatabase follows the structure described in Online-only Table 2. A full description of each individual dataset is reported in the metadata file ‘*DATASET_overview_v2.0_20201030.docx*’, available at the same link.

## Technical Validation

We benchmarked our data against country-level statistics on primary forest extent. Although we had no direct control of the raw data contained in our database, the fact that all our information on primary forest locations derives either from peer-reviewed scientific literature, or was field-checked by trained researchers and/or professionals suggests high data reliability. We made sure to have a common understanding with data contributors about forest definitions [i.e.^34,35^,], and only included a dataset in the EPFD if we could find an explicit equivalence with the forest definitions we used. Additional information on the harmonization process is reported for individual datasets in the metadata accompanying our geodatabase.

An additional, wall-to-wall validation of our database using remotely-sensed information is currently impossible. Remote sensing data only cover the last 35 years, and even if high resolution laser ranging (LIDAR) might become available in the future, at the moment no reliable workflow exists for mapping primary forests from such multi-sensor data. The alternative is field work, which is clearly unfeasible given the huge area covered by our database, the large number of polygons, and the cost and time effort that would be required for a statistically valid ground sample of data. Still, remote sensing data can be helpful for checking whether a patch of primary forest underwent human disturbance after it was delineated and that is why we implemented a semi-automatic procedure based on Landsat satellite-image time series (1985–2018) (see below).

### Benchmarking against country-level statistics

**Fig. 4:**
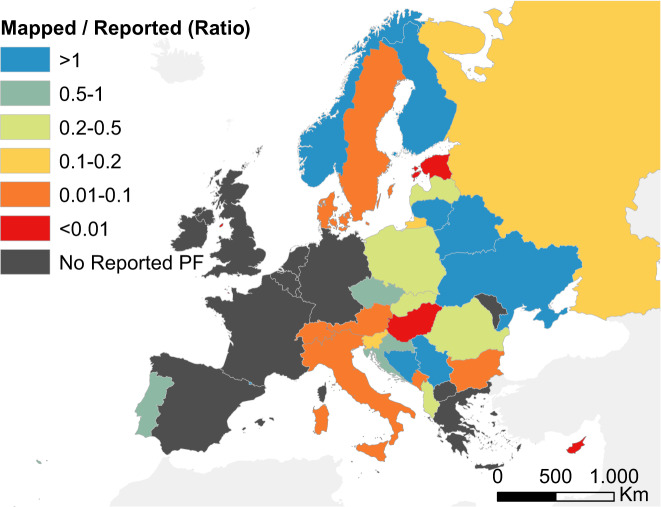
Estimation of data completeness. Ratio between the total primary forest area in the EPFD v2.0 and the country estimate of ‘forest undisturbed by man’ (indicator 4.3) from Forest Europe^10^ or, if unavailable, the country estimates of primary forests based on FAO’s Forest Resources Assessment^48^. Gray polygons represent countries where Forest Europe (or FAO) reports no forest undisturbed by man (‘No Reported PF’).

Our database contains most of the geographical information currently available on primary forests in Europe, but we do not claim this data is complete. To benchmark the completeness of our map, we calculated the ratio between the area of primary forest in our database at country level, and the estimated area of “forest undisturbed by man” from the indicator 4.3 in the Forest Europe report^10^ or, for those countries where this information is not available, from FAO’s Forest Resources Assessment^48^. Although the definition of “forest undisturbed by man” in Forest Europe is consistent with our definition of primary forest^10^, it must be noted that these country-level estimates stem from national inventories or other studies, and data quality varies from country to country^49^. The comparison presented here should, therefore, be taken with caution (Fig. 4).

Forest Europe reports no primary forest for some western European countries (Spain, France, Belgium, Netherlands, Germany, United Kingdom and Ireland), although for most of these countries we did find information on at least a handful of primary forest sites. The coverage of our map was also higher than expected for some Eastern European countries (e.g., Ukraine, Belarus, Lithuania), as well as Norway and Finland, known for hosting large areas of primary forests. Data completeness was lower for some central European countries. In the case of Czechia, Slovakia, Poland and Romania, our data only accounted for 20–100% of the country-level estimates from Forest Europe^10^. For Austria, Switzerland and Hungary, instead, additional data on primary forests exists but it is not currently open-access, and therefore not considered here. The largest data gaps were in Sweden, Italy, Bulgaria, Estonia, Denmark and Russia, where our map accounted for less than 10% of the primary forest reported in Forest Europe^10^. The low data completeness found for Denmark likely depends on the inclusion of minimum-intervention forest reserves in Forest Europe (see^49^) that were harvested until recently and therefore do not qualify as primary forests according to our definition.

### Assessing recent human disturbance with remote sensing

**Fig. 5:**
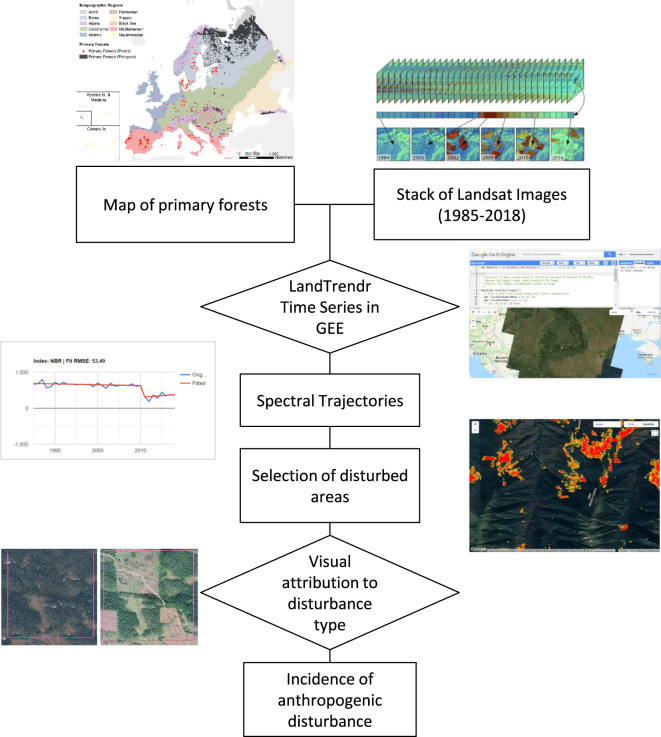
Workflow of the assessment of recent human disturbance in primary forest polygons.

Since our data were collected continuously over the last two decades, we cannot exclude that some forest patches may have undergone human disturbance after data collection. This is particularly relevant for areas where primary forests are lost at high rates, such as the Carpathians, Russian Karelia, or Northern Fennoscandia^18–20^. To assess to what extent this might be an issue, we used the open-access Landsat archive and the LandTrendr disturbance detection algorithm^50,51^, using Google Earth ﻿Engine^52^ (Fig. 5). Specifically, we 1) quantified the proportion of polygons in our map that underwent disturbance between 1985 and 2018, i.e., Landsat 5 operating time, 2) visually checked a stratified random selection of these disturbed polygons to quantify the prevalence of anthropogenic vs. natural disturbance, and 3) estimated the proportion of polygons in our map not meeting the necessary, but not sufficient, condition for being classified as primary (i.e. not being affected by anthropogenic disturbance within the last 35 years).

For each polygon contained in the map of primary forests, we extracted the whole stack of available Landsat images (~1985-today), and ran the LandTrendr^53^ algorithm. LandTrendr identifies breakpoints in spectral time series, separates periods of disturbance or stability, and records the years in which disturbances occurred. To avoid problems due to cloud cover, changes in illumination, and atmospheric condition, we used all available images from the growing season of each year (1 May through 15 September) to derive yearly composite images^54^. As our spectral index, we used Tasseled Cap Wetness (TCW), as this index is particularly sensitive to forest structure^55^, is robust to spatial and temporal variations in canopy moisture^56^, and consistently outperforms other spectral indices, including Normalized Difference Vegetation Index^53^, for detecting forest disturbance^50,57–59^. As input parameters for the LandTrendr algorithm when detecting forest disturbances, we used a prevalue of −300 TCW units, a minimum disturbance magnitude of 500 TCW units, and a maximum duration of 4 years.

**Fig. 6:**
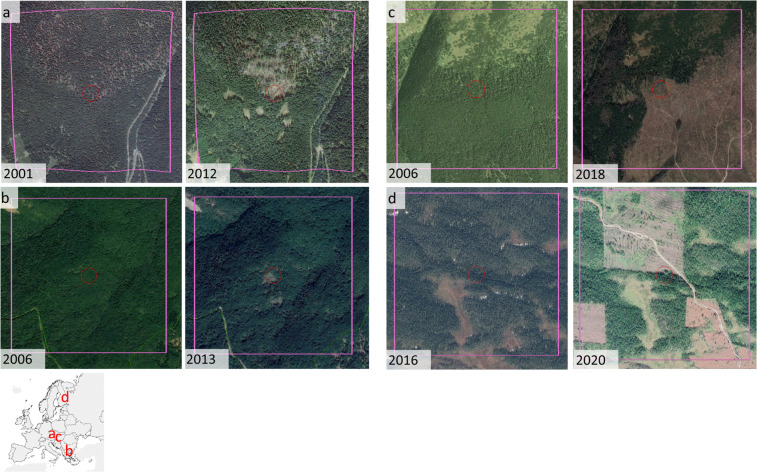
Examples of disturbed polygons, as detected by LandTrendr, before (left) and after (right) disturbance. (**a**) Natural disturbance in Babia Gora, Slovakia; (**b**) natural disturbance in the southern Bourgas Province of Bulgaria; (**c**) clear-cuts in Tatra National Park in Slovakia; (**d**) clearcuts in the Russian Republic of Karelia. Red circles are centred on the disturbed pixel randomly selected for visual inspection, and have a radius of 50 m; pink squares have a side of 1 km and were exclusively used to provide context reference to the photointerpreter. Image credits: Google Earth.

**Fig. 7:**
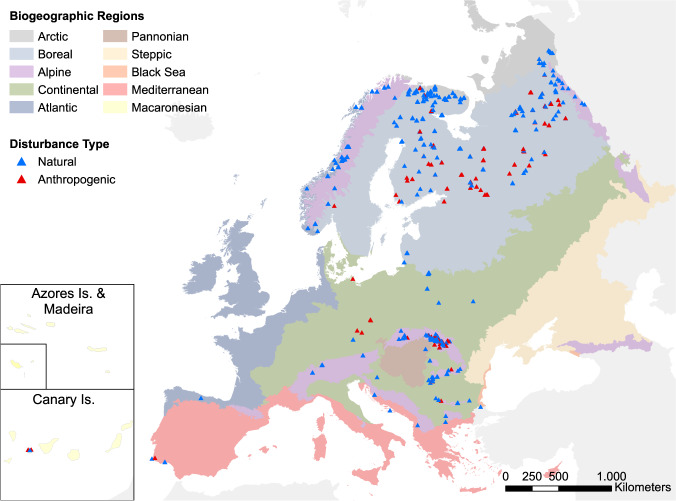
Geographical distribution of naturally vs. anthropogenically disturbed polygons, as resulting from a visual check of 712 pixels across 268 polygons.

After running LandTrendr, we eliminated noise by applying a minimum disturbance threshold (2 ha). We then visually inspected a stratified random selection of primary forest polygons highlighted as ‘disturbed’ by LandTrendr using very-high-resolution images available in Google Earth. For each biogeographic region, we randomly selected 20% of disturbed polygons up to a maximum of 100 polygons per region. Depending on the size of the polygons, we inspected up to 5 randomly selected disturbed pixels within each disturbed polygon with a minimum distance between pixels of 1 km. Based on the spectral and physical characteristics of the disturbed patch (brightness, shape, size), and on ancillary information derived from the Google Earth imagery, we assigned disturbance agents as either anthropogenic (i.e., forest harvest, infrastructure development) or natural (e.g., windstorm, bark beetle outbreak, fire; Figs. 6, 7). We conservatively considered a polygon as anthropogenically disturbed if at least a third of the points we checked for that polygon were anthropogenically disturbed. To avoid introducing an observer bias, all polygons were checked by the same photo-interpreter (FMS).

Out of the 17,309 polygons checked with LandTrendr, 4,734 (27.3% of total) experienced major disturbances between 1985 and 2018. The proportion of disturbed area was greater than 10% in 2,904 polygons. We visually inspected a total of 712 pixels across 268 primary forest polygons, corresponding to 1.5% of the total number of polygons and 5.7% of the disturbed polygons. We attributed a total of 149 pixels, across 61 primary forest polygons, to anthropogenic disturbance, i.e., 22.7% (bootstrapped standard error = 2.5%) of the polygons we checked (Table 2, Fig. 7). We thus estimated the total number of primary forest polygons being anthropogenically disturbed by multiplying the total number of polygons with the proportion of disturbed polygons (27.3%) and the share of these disturbed polygons attributed to anthropogenic causes (22.7%). This suggests our map contains 1,077 anthropogenically disturbed polygons (95% CIs [847, 1323]), which corresponds to 6.2% (95% CIs [4.9%, 7.6%]) of the total number of polygons. Disturbed polygons were concentrated in the Russian Federation (especially in Archangelsk region, Karelia and Komi republics), Southern Finland, and the Carpathians (Fig. 7; Table 2). The Boreal and Alpine biogeographical regions had the highest number of disturbed polygons (both in total, and when considering only those with evident anthropogenic disturbance). The regions with the highest share of anthropogenically disturbed polygons were the Continental and Boreal region. The sample size in Macaronesia was too low to provide a reliable estimation of the incidence of human disturbance.

**Table 2:** Recent human disturbance in primary forest polygons, summarized by biogeographical region.

Biogeographic region	Num. PF polygons (1)	Num. disturbed PF polygons (2)	Num. disturbed PF polygons checked (3)	Num. of (3) with evident anthropogenic disturbance(4)	Share of (3) anthropogenically disturbed (4/3) %
Alpine	11,734	1,096	102	23	22.55
Arctic	96	105^†^	20	0	0
Atlantic	83	48	13	0	0
Black Sea	19	6	1	0	0
Boreal	4,074	3,334	110	30	27.27
Continental	1,100	105	21	6	28.57
Macaronesia	27	8	2	1	50
Mediterranean	132	27	5	1	20
Pannonian	39	4	1	0	0
Steppic	5	1	0	0	0

^†^The number of disturbed polygons is higher than the total number of polygons because some polygons expanding over more than one biogeographical region were split. PF – Primary Forest.

These estimates should be considered as lower bounds, because only the disturbance events with a magnitude sufficient to be captured with LandTrendr and occurring in 1985–2018 could be identified. Not being this a formal validation, the results presented here should not be extrapolated to primary forests not included in our map. Finally, being our database built with a bottom-up approach, we are unable to exclude the existence of remaining bias or interpretation error, which might have propagated through the successive steps required to build it. As such, we warn the users against possible heterogeneity in data quality, accuracy and completeness across datasets.

## Usage Notes

All data files are referenced in a geographic coordinate system (lat/long, WGS 84 - EPSG code: 4326). The provided files are in a personal geodatabase, and can be accessed and displayed using standard GIS software such as: QGIS (www.qgis.org/en).

All datasets listed in Online-only Table 1 are freely available in Figshare (10.6084/m9.figshare.13194095.v1)^33^ with a Creative Commons CC BY 4.0 license. Two additional non open-access datasets are available on request to the corresponding author after approval of the respective copyright holders. These datasets are: ‘Hungarian Forest Reserve monitoring’ (ID 17, custodian: Ferenc Horváth); and ‘Potential OGF and primary forest in Austria’ (ID 48, custodian: Matthias Schickhofer). The same conditions apply for additional data from the dataset ‘Strict Forest Reserves in Switzerland’ (ID 30, custodian: Jonas Stillhard). In the case of the dataset ‘Ancient and Primeval Beech Forests of the Carpathians and Other Regions of Europe’^40,41^ (ID 34, Custodian: UNESCO), this data is freely available online, but its copyright does not allow redistribution. We refer the interested reader to the website https://www.protectedplanet.net/903141 for the original data.

Comments and requests of updates for the dataset are collected and discussed in the GitHub forum: https://github.com/fmsabatini/PrimaryForestEurope.

## Data Availability

The code to reproduce the composite layers, for post-processing and for assessing recent human disturbance with remote sensing is available together with the database in Figshare (10.6084/m9.figshare.13194095.v1)^33^. We included seven scripts: • *00_ComposeMap.R* – Identifies overlapping polygons across individual datasets. • *01_CreateComposite_Points.py* – Creates the composite point feature class. • *02_CreateComposite_Polygons.py* – Creates the composite polygon feature class. • *03_PostProcessing.R* – Extracts additional information on each primary forest. • *04_Add_Postprocessing.py* – Imports post-processing output into the geodatabase. • *05_Summary_stats.R* – Calculates summary statistics of primary forests • *06_DisturbanceAssessment_Step1_exportIntermediateChangeImg.txt* – Runs LandTrendr in Google Earth Engine, tiles the area of interest, creates Change-Images for each tile, and exports these as intermediate .tif files containing the LandTrendr metrics. • *07_DisturbanceAssessment_Step2_extractPolygonValuesFromChangeImg.txt* – Extracts LandTrendr metrics for each forest polygon from Change-Images and exports as .csv. Python (.py) scripts were run in ESRI ArcGIS (v10.5) and are available also as ArcGIS Models inside the Geodatabase. R (.R) scripts were run using R (v 3.6.1)^46^. The remaining .txt scripts were run in Google Earth Engine.

## Online-only Tables

**Online-only Table 1:** Synthetic description of datasets retrieved.

Dataset ID in EPFD v2.0^a^	Dataset name	Custodian	Num. features (Polygons\ Points)	Tot. estimated area (1,000 ha)^b^	Source
0	Literature Review - Primary Forest of Europe	Francesco Maria Sabatini	0\106	85.83*	^31^
1	Forest Ecology Group CULS – REMOTE primary forests	Martin Mikolas	22\0	1.91	^31,38,67,68^
2	LomJanPerBio	Matteo Garbarino & Renzo Motta	4\0	4.45	^31,69–72^
3	WWF - Old-growth forests in Bulgaria	Tzvetan Zlatanov	129\0	51.93	^31^, https://gis.wwf.bg/mobilz/en/
4	Coniferous Old-growth forest of Rila and Pirin NP, Bulgaria	Momchil Panayotov	363\0	3.3	^31,73^
5	Croatian OG forests reserve	Stjepan Mikac	46\0	7.28	^31^
6	Czech natural forests databank	Dušan Adam; Tomas Vrska	86\0	8.17	^31^, https://naturalforests.cz/
7	Old-growth & long untouched forests of Denmark	Erik Buchwald	0\24	1.68	^31^
8	Hemiboreal old-growth forests of Estonia	Ann Kraut	0\23	0.05	^31,74^
9	High Value Beech Forest in Europe	Fabio Lombardi	0\10	0.57	^31,75^
12	Publicly available data on OG forests of Finland	Olli-Pekka Tikkanen	681\0	2740.5	^31^. Derived from https://www.syke.fi/en-US/Open_information
13	WWF - Hauts lieux de naturalité en France	Daniel Vallauri	49\0	0.19	^31,76^, http://www.foretsanciennes.fr/proteger-mieux/france/
14	RNF	Eugénie Cateau	7\0	5.31	^31,37^
15	Naturwaldreservate & Weltnaturerbe Buchenwälder in Deutschland	Peter Meyer	24\7	5.81*	^31^, https://fgrdeu.genres.de/naturwaldreservate
16	World Heritage Beech Forests of Europe - Greek candidates	Nikolaos Grigoriadis	5\0	1.75	^31^
18	Old-growth forests in Italian National Parks	Sabina Burrascano	67\0	3.58	^31,36^ & Unpublished
19	Long-untouched forests in Lithuania	Gintautas Mozgeris	20\0	32.05	^31^
20	PriMaFor - Primary forests in Mavrovo NP	Bojan Simovski	4\1	0.68	^31^
21	Old-growth forests in Norway outside protected areas	Rein Midteng	50\0	106.29	^31,77^
22	Database of old-growth forests of Poland	Jerzy Szwagrzyk	66\0	20.87	^31^
23	Natural forest areas in Portugal	Inês Marques Duarte	31\21	1.11*	^31^
24	Natural forest areas in Portuguese Macaronesia region^‡^	Leónia Nunes	1\0	14.64	^31^
25	WWF - Lemnocontrolat	Radu Melu	3179\0	46.68	^31^, http://www.lemncontrolat.ro
26	PRALES Database	Juraj Vysoky	290\0	10.58	^19,31^, http://en.pralesy.sk
27	Graficni prikaz gozdnih rezervatov	Rok Pisek	170\0	9.51	^31^, http://www.zgs.si/gozdovi_slovenije/ o_gozdovih_slovenije/gozdni_rezervati/index.html
29	Dynamic edge effects on Boreal forest	Alejandro Ruete	0\31	0.97	^31,78^
30	Strict Forest Reserves in Switzerland	Jonas Stillhard	5\0	0.73	^31,79,80^
32	WWF - Identified old-growth forests of Ukrainian Carpathians and Polissia	Roman Volosyanchuk, Andriy Plyha	9068\0	97.86	^31^, http://gis-wwf.com.ua/
33	Official Romanian catalogue of virgin and quasi-virgin forests	Romanian Ministry of Forest and Waters	1287\0	19	https://www.inspectorulpadurii.ro
35	European Beech Forest Network (EBFN) sites	Marcus Waldherr, Pierre Ibisch	0\32	28.29*	Unpublished
36	OGF Collection from Netherlands, Serbia and Norway	Francesco Maria Sabatini	8\0	29.48	NL 10.17026/dans-2bd-kskz RS^81^ NO - https://www.environmentagency.no/
37	Inventory of both ancient and mature forests on the northern slope of the Pyrenees_GEVFP	Laurent Larrieu	51\0	3.25	^82,83^
38	Kainuun vanhat metsät	Matti Liimatainen	123\0	6.43	Unpublished
39	Kansallisomaisuus turvaan	Paloma Hannonen	204\0	71.18	^84^
40	Natural forests in Latvia	Mara Kitenberga	3\0	4.79	^85–87^
41	Garajonay	Ángel B. Fernández López	85\0	2.4	^88–90^
43	Foreslåtte verneområder	Rein Midteng	200\0	196.73	https://biofokus.no/narin/
44	Serbia Beech OGF	Bratislav Matović; Dejan Stojanović	5\0	0.15	^91^
45	Protected virgin & old growth forests in the Pannonian biogeographical region in Serbia	Alen Kiš	8\0	0.65	^92^
46	Forest-mire ecosystems in Belovezhskaya pushcha National Park, Berezinski biosphere reserve, Olmany reserve in Belarus	Maxim Yermokhin	3\0	188.29	Unpublished
47	Albanian Primary Forests	Elvin Toromani	0\4	0.65	Unpublished
49	Suspected Primeval Forests of the Kalkalpen Nationalpark	Simone Mayrhofer	34\0	0.45	Unpublished
50	VF Montenegro	Stjepan Mikac	2\0	1.65	Unpublished
51	Primary Forests of European Russia	Dmitry Aksenov; Asiya Zagidullina	3084\0	37417.69	^13,39,93^
52	Red de Rodales de Referencia (Network of Reference Stands)	Jose A. Atauri	0\54	0.89	http://www.redbosques.eu/
53	Primary forests in Bosnia	Srđan Keren	0\9	0.72	Unpublished
54	Old beech forests in Albania	Abdulla Diku	13\0	12.7	^94^
55	Network of old-growth forests in southern Apennine National Parks	Sabina Burrascano	19\0	2.78	^95^

^a^ID codes correspond to the numbering in the geodatabase, and correspond to the numbering used in EPFD v1.0^31^, for those datasets which are shared between v1.0 and v2.0. Some IDs are missing, either because the IDs were originally assigned to datasets which were successively replaced by or merged with others, or because they correspond to datasets which are not open-access (IDs 17, 24, 48), and therefore not presented here. Additional information on non-open-access datasets is provided in the Usage notes. ^b^Forest areas reported in this table do not account for overlaps across different datasets. For applications having a regional or continental scope, users are recommended to use the composite feature classes described in the section ‘Data records’, which only include unique non-overlapping points and polygons. *Some point features have no information on forest patch area.

^‡^this dataset belongs to the Regional Forest Service of Madeira.

**Online-only Table 2:** Spatial attributes of the feature classes of primary forests.

Variable Name	Variable_type	Description and possible values
OBJECTID	Object ID	
FOREST_NAME	Text	Name of the forest stand (if applicable, otherwise can be name of the wider area)
LOCATION	Text	Municipality, Protected Area, or Region in which the primary forest remnant is located
NATURALNESS_LEVEL	Short Integer	Naturalness level according to^35^: Possible values: 10 = n10 - Primeval Forest; 9 = n9 - Virgin Forest; 8 = n8 - Frontier Forest; 7 = n7 - Near-virgin Forest; 6 = n6 - Old-growth Forest; 5 = n5 - Long Untouched Forest; 0 = UNKNOWN
FOREST_EXTENT_MEASURED^‡^	Float	The total extent of the primary forest patch in hectares. This field is only relevant when a polygon feature IS NOT available for the forest patch.
FOREST_EXTENT_ESTIMATED^‡^	Short Integer	The order of magnitude of the extent of a primary forest remnant patch. This field is only relevant when a polygon feature IS NOT available for the forest patch and no precise measurement of the total extent of the forest remnant is available. Possible values: 1 = 1–10 ha; 2 = 11–100 ha; 3 = 101–1000 ha; 4 = > 1001 ha
DOMINANT_TREE_SPECIES1	Text	Species (latin name) of the dominant tree species of the overstorey
DOMINANT_TREE_SPECIES2	Text	Species (latin name) of the second dominant tree species of the overstorey (if any)
DOMINANT_TREE_SPECIES3	Text	Species (latin name) of the third dominant tree species of the overstorey (if any)
THREAT_1	Short Integer	Threat (if any) that is most likely to endanger the primary forest remnant. Possible values: 1 = Plantation development; 2 = Anthropogenic Fires; 3 = Tourism/recreation; 4 = Infrastructure development (including touristic); 5 = Mismanagement; 6 = Illegal logging; 7 = Timber and fuelwood extraction; 8 = Non-Timber Forest Products extraction; 9 = Urbanization and housing construction; 10 = Climate change; 11 = Biodiversity loss
THREAT_2	Short Integer	Threat (if any) that is most likely to endanger the primary forest remnant. See above for possible values.
LAST_DISTURBANCE1_TYPE	Text	If known, type of the last disturbance event. Possible values: 1 = Fire, 2 = Windthrow; 3 = Flood; 4 = Landslide Avalanche; 5 = Logging\harvesting; 6 = Diseases\insect outbreak; 7 = OTHER natural; 8 = OTHER anthropogenic
LAST_DISTURBANCE1_YEAR	Short Integer	Year when disturbance event 1 happened
LAST_DISTURBANCE1_INTENSITY	Short Integer	Intensity of disturbance event 1. Possible values: 1 = Light (<20% of the stand disturbed); 2 = Moderate (20–70% of the stand disturbed); 3 = Stand replacing (>70% of the stand disturbed)
LAST_DISTURBANCE2_TYPE	Text	If known, type of the penultimate disturbance event Possible values: see above
LAST_DISTURBANCE2_YEAR	Short Integer	Year of disturbance event 2
LAST_DISTURBANCE2_INTENSITY	Short Integer	Intensity of disturbance event 2 – Possible values: see above
PROTECTION_STATUS	Short Integer	Legal protection status of the forest stand as derived from the World Database of Protected. The original IUCN classification was simplified to three classes: Strictly protected (IUCN category I); Protected (IUCN categories II-VI + not classified); Not protected. In case more updated/precise information was available from our data contributors, these were given priority. Possible values: 0 = Not protected; 1 = Protected; 2 = Strictly protected
PROTECTED_SINCE	Short Integer	Year since the onset of legal protection, derived the same way as PROTECTION_STATUS, see above
RELEVANT_LITERATURE	Text	Any relevant sources of information describing the forest remnant (including journal articles, local reports and websites)
CONTACT_PERSON	Text	Name of the contact person providing the information on the stand
Notes	Text	optional additional remarks to the forest polygon
Source	Text	Directly attributable source/ownership attribution of the forest remnant data
ID_Dataset	Text	ID of the data set (as in Online-only Table 1)
Priority	Integer	An integer number describing the priority of the polygon in case of overlap across individual datasets. For polygons of lower priority, only the portion of polygon not overlapping with polygons with higher priority was included in the composite dataset. Polygons with priority = 99 were not included in the composite dataset
Area_ha	Float	area of the forest polygon in ha
BIOGEOGRAPHIC_REGION	Text	as defined by the European Environmental Agency^42^
FOREST_TYPE1	Short Integer	Main forest type according to the forest categories defined by the European Environmental Agency^43^, based on the map of Potential Vegetation type for Europe^21^. Possible values: 1 = Boreal; 2 = Hemiboreal-nemoral; 3 = Alpine coniferous; 4 = Acidophilus oak-birch; 5 = Mesophytic deciduous; 6 = Lowland beech; 7 = Montane beech; 8 = Thermophilus deciduous; 9 = Broadleaved evergreen: 10 = Coniferous Mediterranean; 11 = Mire and swamp; 12 = Floodplain; 13 = Non-riverine Alder-birch-aspen
FOREST_TYPE2	Short Integer	Second main forest type according to the forest categories defined by the European Environmental Agency^43^, based on the map of Potential Vegetation types for Europe^21^. See FOREST_TYPE1 for legend
FOREST_SHARE	Float	Actual share of the polygon covered by forest, assuming that primary forests in high naturalness classes, and having a large extent, may encompass land temporarily or permanently not covered by forest. Derived from high resolution maps of forest cover based on ^45^.

^‡^Only for point feature classes.
