# Author Correction: European primary forest database v2.0

**DOI:** 10.1038/s41597-021-01036-0

**Published:** 2021-09-20

**Authors:** Francesco Maria Sabatini, Hendrik Bluhm, Zoltan Kun, Dmitry Aksenov, José A. Atauri, Erik Buchwald, Sabina Burrascano, Eugénie Cateau, Abdulla Diku, Inês Marques Duarte, Ángel B. Fernández López, Matteo Garbarino, Nikolaos Grigoriadis, Ferenc Horváth, Srđan Keren, Mara Kitenberga, Alen Kiš, Ann Kraut, Pierre L. Ibisch, Laurent Larrieu, Fabio Lombardi, Bratislav Matovic, Radu Nicolae Melu, Peter Meyer, Rein Midteng, Stjepan Mikac, Martin Mikoláš, Gintautas Mozgeris, Momchil Panayotov, Rok Pisek, Leónia Nunes, Alejandro Ruete, Matthias Schickhofer, Bojan Simovski, Jonas Stillhard, Dejan Stojanovic, Jerzy Szwagrzyk, Olli-Pekka Tikkanen, Elvin Toromani, Roman Volosyanchuk, Tomáš Vrška, Marcus Waldherr, Maxim Yermokhin, Tzvetan Zlatanov, Asiya Zagidullina, Tobias Kuemmerle

**Affiliations:** 1 German Centre for Integrative Biodiversity Research (iDiv) - Halle-Jena-Leipzig, Puschstrasse 4, 04103 Leipzig, Germany; 2 Martin-Luther-Universität Halle-Wittenberg, Institut für Biologie, Am Kirchtor 1, 06108 Halle, Germany; 3 Humboldt-Universität zu Berlin, Geography Department, Unter den Linden 6, 10099 Berlin, Germany; 4 Frankfurt Zoological Society, Bernhard-Grzimek-Allee 1, 60316 Frankfurt, Germany; 5 NGO “Transparent World”, Rossolimo str. 5/22, building 1, 119021 Moscow, Russia; 6 EUROPARC-Spain/Fundación Fernando González Bernáldez. ICEI Edificio A. Campus de Somosaguas, E28224 Pozuelo de Alarcón, Spain; 7 The Danish Nature Agency, Gjøddinggård, Førstballevej 2, DK-7183 Randbøl, Denmark; 8 Sapienza University of Rome, Department of Environmental Biology, P.le Aldo Moro 5, 00185 Rome, Italy; 9 Réserves Naturelles de France, La Bourdonnerie, Dijon cedex, 21000 France; 10 PSEDA-ILIRIA. Forestry department, Tirana, 1000 Albania; 11 Centre for Applied Ecology “Professor Baeta Neves” (CEABN), InBIO, School of Agriculture, University of Lisbon, Tapada da Ajuda, 1349‐017 Lisbon, Portugal; 12 Parque Nacional de Garajonay. Avda. V Centenario, edif. Las Creces, local 1, portal 3, 38800 San Sebastian de La Gomera, Tenerife, Spain; 13 University of Torino, Department DISAFA L.go Paolo Braccini 2, Grugliasco, 10095 Italy; 14 Forest Research Institute, Vassilika, 57006 Thessaloniki, Greece; 15 Centre for Ecological Research, Institute of Ecology and Botany, Alkotmány u. 2-4, 2163 Vácrátót, Hungary; 16 Faculty of Forestry, University of Agriculture in Krakow, aleja 29-Listopada 46, 31-415 Krakow, Poland; 17 Latvian State Forest Research Institute “Silava”, Rigas street 111, Salaspils, LV-2169 Latvia; 18 Institute for Nature Conservation of Vojvodina Province, Radnička 20a, Novi Sad, 21000 Serbia; 19 University of Tartu, Institute of Ecology and Earth Sciences, Vanemuise 46, EE-51014 Tartu, Estonia; 20 Centre for Econics and Ecosystem Management, Faculty of Forest and Environment, Eberswalde University for Sustainable Development, Alfred-Möller-Str. 1, 16225 Eberswalde, Germany; 21 Université de Toulouse, INRAE, UMR DYNAFOR, 24 Chemin de Borde-Rouge Auzeville CS 52627, Castanet-Tolosan, 31326 France; 22 CRPF-Occitanie, antenne de Tarbes, place du foirail, 65000 Tarbes, France; 23 Mediterranean University of Reggio Calabria, Agraria Department, Loc. Feo di Vito, 89122 Reggio Calabria, Italy; 24 University of Novi Sad, Institute of Lowland Forestry and Environment, Antona Cehova 13d, Novi Sad, 21102 Serbia; 25 World Wide Fund for nature (CEE), Lunga street 190, Brasov, 500051 Romania; 26 Northwest German Forest Research Institute, Department Forest Nature Conservation, Professor-Oelkers-Straße 6, 34346 Hann. Münden, Germany; 27 Asplan Viak A.S.Kjörboveien 20, postboks 24, N-1300 Sandvika, Norway; 28 University of Zagreb, Faculty of Forestry, Svetosimunska cesta 25, 10000 Zagreb, Croatia; 29 Czech University of Life Sciences, Faculty of Forestry and Wood Sciences, Kamýcka cesta 1176, CZ-, 16521 Praha6-Suchdol, Czech Republic; 30 PRALES, Odtrnovie 563, SK-01322 Rosina, Slovakia; 31 Vytautas Magnus University, K. Donelaičio g. 58, LT-44248 Kaunas, Lithuania; 32 University of Forestry, Dendrology Department, bulevard “Sveti Kliment Ohridski” 10, 1756 Sofia, Bulgaria; 33 Slovenia Forest Service, Department for forest management planning, Vecna pot 2, 1000 Ljubljana, Slovenia; 34 Centre for Applied Ecology “Professor Baeta Neves” (CEABN), InBIO, School of Agriculture, University of Lisbon, Tapada da Ajuda 1349‐017, Lisbon, Portugal; 35 Greensway AB, Ulls väg 24A, 756 51 Uppsala, Sweden; 36 Freelance forest expert and book author, Vienna, Austria; 37 Ss. Cyril and Methodius University in Skopje, Hans Em Faculty of Forest Sciences, Landscape Architecture and Environmental Engineering, Department of Botany and Dendrology, P.O. Box 235, MK-1000 Skopje, North Macedonia; 38 Swiss Federal Research Institute for Forest, Snow and Landscape Research WSL, Forest Resources and Management, Zürcherstrasse 111, 8903 Birmensdorf, Switzerland; 39 University of Novi Sad, Institute of Lowland Forestry and Environment, Antona Cehova 13d, Novi Sad, 21000 Serbia; 40 Department of Forest Biodiversity, University of Agriculture, Kraków, Poland; 41 University of Eastern Finland, School of forest Sciences, Yliopistokatu 7, 80100 Joensuu, Finland; 42 Agricultural University of Tirana, Forestry Department, Kodër Kamëz, SH1, 1029 Tirana, Albania; 43 World Wide Fund for nature (DCP) Ukraine, Mushaka 48, Lviv, 79011 Ukraine; 44 Ecosphera NGO, Kapushans’ka 82a, Uzhhorod, 88000 Ukraine; 45 Silva Tarouca Research Institute, Department of Forest Ecology, Lidická 25/27, 602 00 Brno, Czech Republic; 46 Centre for Econics and Ecosystem Management, Faculty of Forest and Environment, Eberswalde University for Sustainable Development, Alfred-Möller-Str. 1, 16225 Eberswalde, Germany; 47 Institute of Experimental Botany of the National Academy of Sciences of Belarus, Laboratory of Productivity & Stability of Plant Communities, 220072, Academicheskaya St. 27, Minsk, Belarus; 48 Institute of Biodiversity and Ecosystem Research, Bulgarian Academy of Sciences, 2 Gagarin Street, 1113 Sofia, Bulgaria; 49 Saint-Petersburg State University, Department of Vegetation Science, University Embankment, 7/9, St Petersburg, 199034 Russia; 50 Humboldt-Universität zu Berlin, Geography Department & Integrative Research Institute on Transformation in Human-Environment Systems, Unter den Linden 6, 10099 Berlin, Germany

**Keywords:** Biodiversity, Forest ecology, Conservation biology

Correction to: *Scientific Data* 10.1038/s41597-021-00988-7, published online 17 August 2021

The original version of this Data Descriptor omitted the following information from the Acknowledgements:

This study was realized and funded by the project “Policy and on-ground action for primary forest protection, boreal and temperate primary forests” funded through the Griffith University (Australia) and implemented by the Frankfurt Zoological Society (FZS) and Wild Europe Initiative as well as the Naturwald Akademie. Additional funding derive from the European Commission (Marie Sklodowska‐Curie fellowship to FMS, project FORESTS & CO, #658876).

This has now been corrected in both the PDF and HTML versions of the Data Descriptor.

